# Global burden and trends of adverse effects of medical treatment, 1990–2021: an analysis from the global burden of disease study 2021

**DOI:** 10.3389/fphar.2025.1655864

**Published:** 2025-09-12

**Authors:** Keqing Zhang, Yuhai Gao, Yunfei Lu

**Affiliations:** ^1^ Department of Neurology, Zhejiang Hospital, Hangzhou, Zhejiang, China; ^2^ Brain Center, Zhejiang Hospital, Hangzhou, Zhejiang, China; ^3^ Integrated Traditional Chinese and Western Medicine Ward, Zhejiang Hospital, Hangzhou, Zhejiang, China

**Keywords:** adverse effects of medical treatment, incidence, prevalence, mortality, DALYs, health inequality

## Abstract

**Background:**

Adverse effects of medical treatment (AEMT) pose a significant global health concern, yet prior studies have mostly focused on specific adverse events or single countries, leaving the long-term global epidemiological patterns insufficiently characterized. As healthcare utilization grows, it is crucial to comprehensively quantify the global, regional, and national burden of AEMT and to forecast future trends for effective resource allocation and quality improvement.

**Methods:**

We utilized data from the Global Burden of Disease (GBD) study 2021. AEMT were defined based on GBD criteria, and incidence, prevalence, deaths, and disability-adjusted life years (DALYs) data were extracted. This study was stratified by age, gender, region and socio-demographic index (SDI), and estimated annual percentage change was used to assess the trends from 1990 to 2021. Based on SDI, we conducted health inequality analysis and used the Bayesian age-period-cohort model to predict the trend changes over the next 15 years.

**Results:**

Globally, there were 12,481,276 new cases of AEMT, with 122,330 deaths, resulting in 4,846,981 DALYs loss in 2021. The age-standardized incidence (ASIR) and prevalence rates (ASPR) worldwide were showing an upward trend, especially in high SDI regions. Both age-standardized mortality (ASMR) and DALYs rates (ASDR) showed a gradual decline during the study period, but they still carried a heavy burden in the low SDI regions (2021 ASDR: 3.71 [95% UI: 2.90 to 5.68] per 100,000 persons-year; 2021 ASR for DALYs: 150.37 [95% UI: 109.08 to 215.24] per 100,000 persons-year). Australasia demonstrated the highest ASIR and ASPR, while Western Sub-Saharan Africa showed the highest ASMR and ASDR. Health inequality analyses revealed that both absolute and relative inequalities of DALYs were narrowing. By 2036, it is forecast that ASIR will decrease to 72.33 (19.40–125.27) per 100,000 persons-year, and ASDR will decrease to 40.98 (21.52–60.43) per 100,000 persons-year.

**Conclusion:**

This study provided a comprehensive global, regional, and national assessment of the burden and inequality of AEMT over the past three decades, coupled with forecasts to 2036. The findings revealed distinct epidemiological patterns across SDI levels and regions, filling an important knowledge gap and offering evidence to guide healthcare safety strategies, medical education, and surveillance systems to further reduce the burden of AEMT worldwide.

## 1 Introduction

Adverse effects of medical treatment (AEMT) are injuries to patients caused not by the disease itself but by the medical process. AEMT may lead to prolonged hospital stays, disability, and even death (Kamath et al., 2024). The concept of AEMT was first proposed by the Harvard Medical School’s 1984 Practical Study (Brennan et al., 1991). A systematic study showed that the median incidence rate of AEMT was 9.2%, with 43% of cases considered preventable and 7.4% resulting in fatal outcomes (De Vries et al., 2008). Therefore, reducing the incidence of medical-related adverse events and improving the quality of patient care is of great significance to medical health.

A 1999 Harvard study estimated that 44,000 to 98,000 people die annually in the United States due to medical adverse events, introducing the landmark concept “To Err is Human: Building a Safer Health System” (Kohn et al., 2000). This report played a pivotal role in raising awareness and catalyzing patient safety initiatives. In recent years, as people’s attention to AEMT has increased, the burden of AEMT has decreased. More recent national-level analyses, such as those from India, have reported slight declines in mortality attributable to AEMT—for example, from 2.34 per 100,000 in 2010 to 2.33 in 2019. However, these estimates may be affected by underreporting, missing data, and variability in surveillance systems (Landrigan et al., 2010; Classen et al., 2011). Data acquisition often depends on case-based adverse event reporting, which is resource-intensive and prone to reporting bias, particularly in settings where healthcare workers may underreport events due to self-protection concerns or institutional pressures (Hayward and Hofer, 2001; Hibbert et al., 2016). To address this, some countries have established feedback systems. For example, the Indian government has set up a National Hospitals and Healthcare Provider Certification Commission to record the incidence of adverse medical events. The Department of Health and Human Services (DHHS) of the United States collects data on the incidence of adverse events. These data show that adverse medical events remain a major problem (Fujiwara et al., 2024). However, the actual incidence of medical-related adverse events is likely underestimated. This is due to factors such as cultural differences among countries and regions, variability in data collection quality, and doctors’ tendency toward self-protection.

AEMT represents a major public health challenge, but most prior research has important limitations. Earlier studies were largely based on single-country data, hospital registries, or specific clinical settings, which restricted comparability across regions and time periods. In addition, many investigations focused only on selected categories of adverse events (for example, surgical complications or medication errors), overlooking the broader spectrum of AEMT. Importantly, few analyses have systematically examined long-term global, regional, and national trends, health inequalities across socio-demographic contexts, or provided forecasts of the future burden. These limitations hindered a comprehensive understanding of the true global impact of AEMT.

To address these gaps, the present study utilizes data from the Global Burden of Disease Study (GBD) 2021, which offers harmonized definitions, standardized data sources, and advanced modeling techniques. This enables us to provide a comprehensive assessment of the incidence, prevalence, mortality, and disability-adjusted life years (DALYs) attributable to AEMT across 204 countries and territories from 1990 to 2021, with stratification by age, sex, region, and socio-demographic index (SDI). Furthermore, we apply health inequality analyses and Bayesian age–period–cohort modeling to forecast trends through 2036. This study therefore represents the global, systematic, and forward-looking evaluation of the burden of AEMT.

## 2 Materials and methods

### 2.1 Data sources and data collection

The information utilized in this research was sourced from the Global Burden of Disease Study 2021, which is managed by the Institute for Health Metrics and Evaluation (IHME). GBD 2021 integrates demographic and health metrics from 204 countries and territories spanning 1990 to 2021. We accessed the Global Health Data Exchange (GHDx) platform to extract annual estimates of AEMT incidence, prevalence, mortality and disability-adjusted life years (DALYs) (http://ghdx.healthdata.org/gbd-results-tool) (Diseases and Injuries, 2024). The definition of AEMT is based on the ninth and the tenth revisions (Agarwal et al., 2023) of the International Classification of Diseases (ICD) coding, and is referenced from previously published literature (Diseases and Injuries, 2024).

### 2.2 Socioeconomic status

The socio-demographic index (SDI) serves as a composite metric ranking national development levels across populations. This standardized metric ranges from 0 (least developed) to 1 (most developed), derived through min-max normalization of constituent indicators. SDI is based on three indicators: (Kamath et al., 2024): lag-distributed income *per capita*; (Brennan et al., 1991); educational attainment for people aged 15 and above and (De Vries et al., 2008) total fertility rate for women aged under 25. In this study, we classified regions and countries into five quintiles of SDI.

### 2.3 Health inequality analysis

To assess health disparities across socioeconomic groups, the study compared rate differences among different SDI strata. The slope index of inequality (SII) and the concentration index (CI) were applied to measure health inequities.

### 2.4 Bayesian age-period-cohort (BAPC) prediction

Using data spanning 1990 to 2021, we applied an age-period-cohort model to forecast AEMT-related burden trends through 2036. To quantify estimation uncertainty, we employed the Markov chain Monte Carlo (MCMC) algorithm, generating 95% uncertainty intervals (UIs) for the projections.

### 2.5 Statistical analysis

All rates were standardized according to the GBD 2021 standard population and expressed as per 100,000 persons-year. Estimated annual percentage change (EAPC) was calculated to evalute epidemiological trends from 1990 to 2021. All analyses accounted for GBD estimation uncertainty by incorporating 1,000 draw-level replicates in variance calculations. All estimates included 95% uncertainty intervals (95% UIs) with statistical significance threshold at p < 0.05.

### 2.6 Ethics statement

The information used in this research was sourced from GBD 2021, with approval from the University of Washington’s Institutional Review Board. The GBD study strictly adheres to the policies and procedures of The University of Washington and the pertinent federal, state, and local legislation. Therefore, no additional ethical approval or informed consent procedures were necessary for this study. All ethical standards are confirmed through the proper citation of relevant sources.

### 2.7 Patient and public involvement

Patients and/or the public were not involved in the design, or conduct, or reporting, or dissemination plans of this research.

## 3 Results

### 3.1 Global burden of AEMT

**FIGURE 1:**
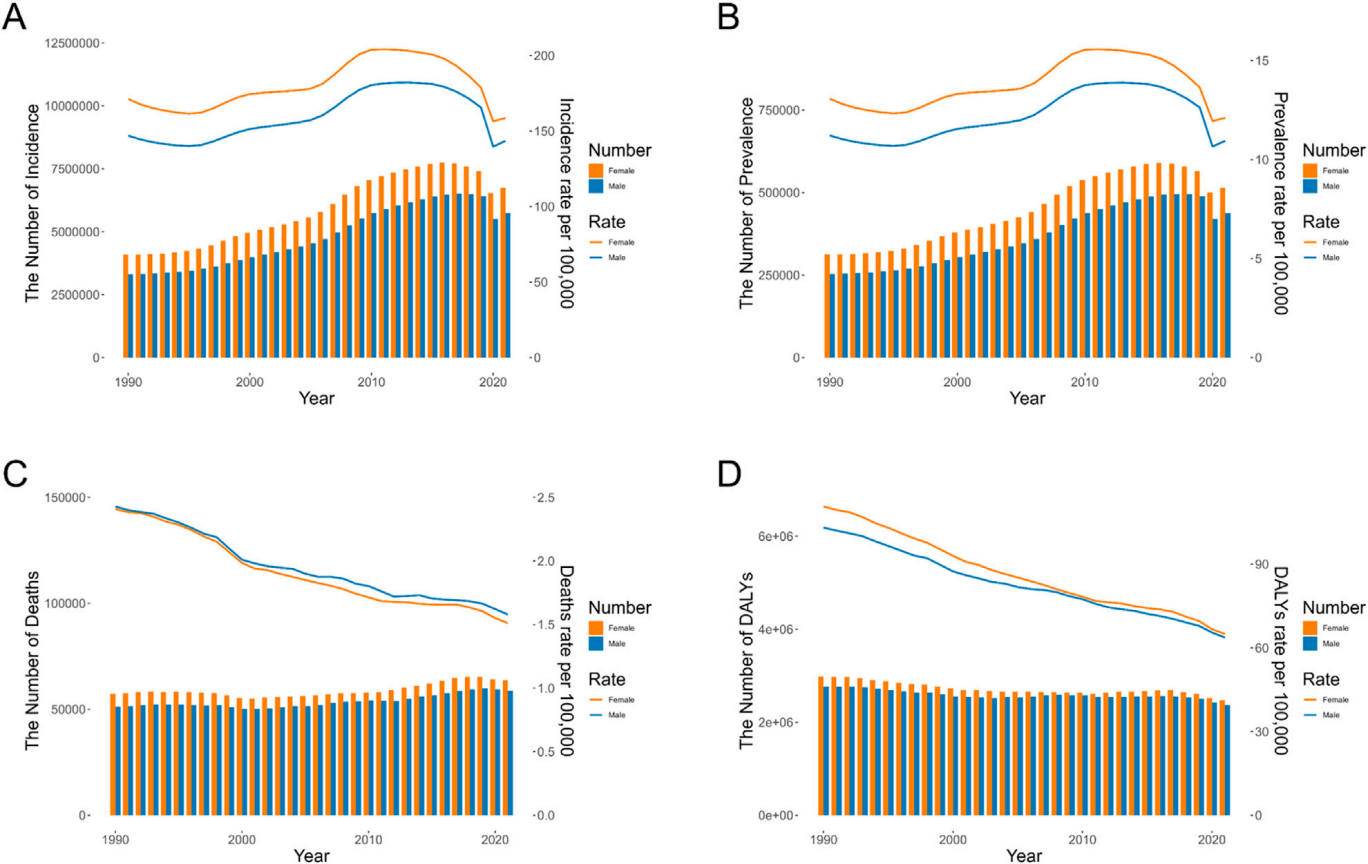
The Number and Age-Standardized Rate of the Global Burden of Adverse effects of medical treatment. **(A)** Incidence. **(B)** Prevalence. **(C)** Deaths. **(D)** DALYs. DALYs: Disability-Adjusted Life Years.

In 2021, there were an estimated 12.48 million (95% UI: 10.89-14.29 million) new cases and 0.95 million (95% UI: 0.73-1.18 million) prevalent cases of AEMT worldwide. Both incidence and prevalence rates peaked in 2010 (191.65 and 14.61 per 100,000, respectively), declined notably in 2020 (147.60 and 11.26 per 100,000), and rebounded slightly in 2021 (150.44 and 11.48 per 100,000). Overall, women had higher rates than men. The highest incidence was observed in females in 2016 (7.74 million cases), while males reached their peak in 2017 (6.50 million cases) (Figures 1A,B).

Globally, there were 122,330 deaths and 4,846,981 DALYs loss attribute to AEMT in 2021. The age-standardized mortality rates (ASMR) and DALY rates (ASDR) were 1.53 (95% UI: 1.29-1.68) and 64.19 (95% UI: 51.06-73.11) per 100,000 population in 2021, respectively. Both mortality and DALYs rates showed a gradual decline over time, with males having slightly higher mortality rates than females, but lower DALYs rates than females. The total number of deaths for both men and women peaked in 2019. Changes in the number of DALYs were more modest, and the differences between men and women were relatively small (Figures 1C,D).

### 3.2 Regional burden of AEMT

**FIGURE 2:**
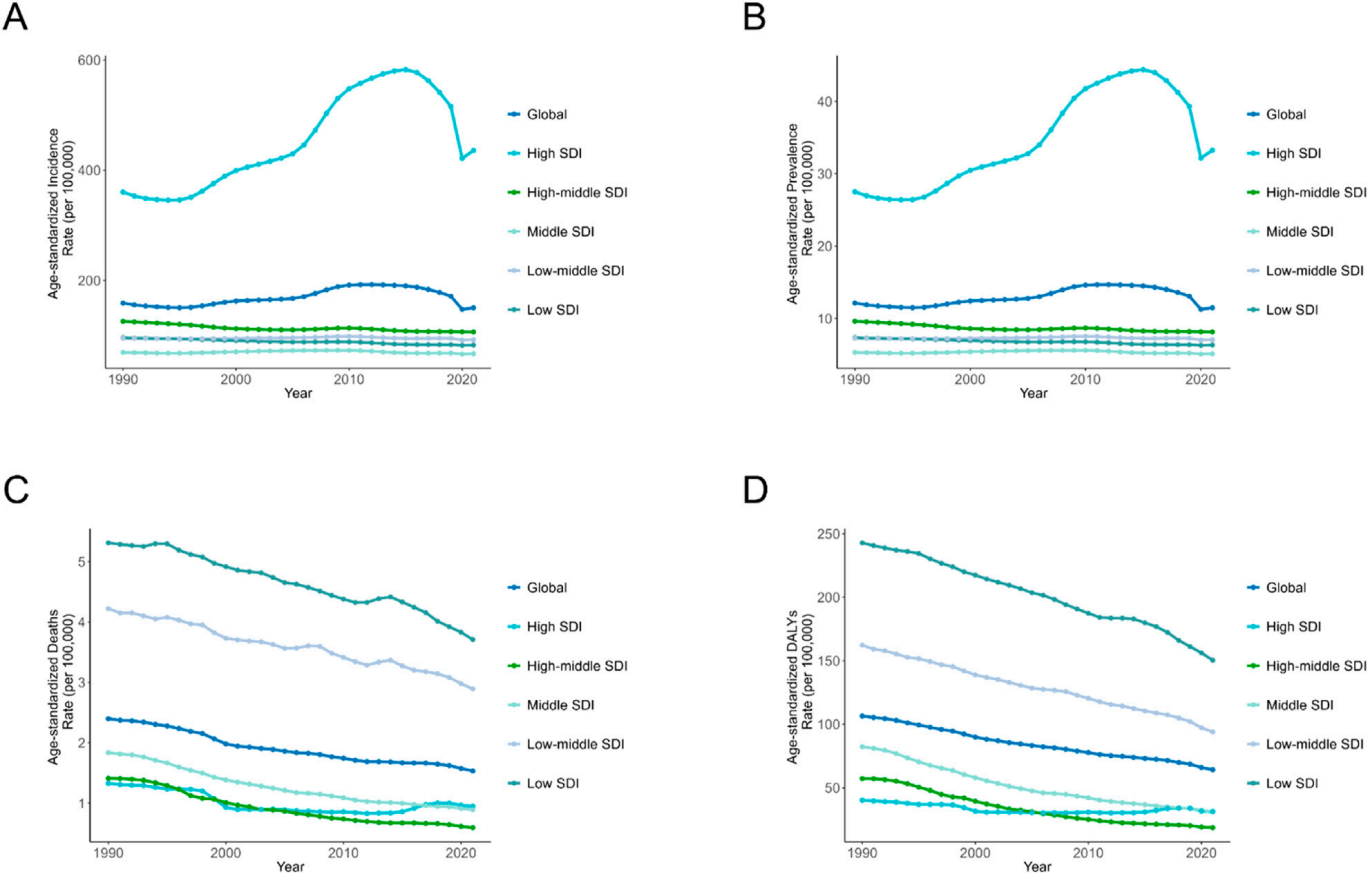
Age-Standardized Rates of Incidence, Prevalence, Deaths, and DALYs Due to Adverse effects of medical treatment, by SDI Quintiles for Both Sexes, 1990–2021. **(A)** Incidence. **(B)** Prevalence. **(C)** Deaths. **(D)** DALYs. DALYs: Disability-Adjusted Life Years; SDI: Socio-Demographic Index.

We found that both ASIR and ASPR of AEMT in high SDI region were significantly higher than the global average, which were 435.86 (95% UI: 377.41, 501.54) and 33.25 (95% UI: 25.54, 41.37), respectively. Middle SDI region had the lowest levels of ASIR and ASPR. Both ASIR and ASPR in high SDI region peaked in 2015, gradually declined in 2016-2020 and slightly recovered in 2021 (Figures 2A,B).

ASDR and ASMR in both low SDI and low-middle SDI regions were significantly higher than the global average, with high-middle regions having the lowest ASDR and ASMR levels in the last 15 years. Specifically, the ASMR and ASDR in low SDI regions were 3.71 (95% UI: 2.90, 5.68) and 150.37 (95% UI: 109.08, 215.24) per 100,000 persons-year, respectively. At the global level, ASDR and ASMR show a slow decreasing trend from 1990 to 2021 (Figures 2C,D).

**FIGURE 3:**
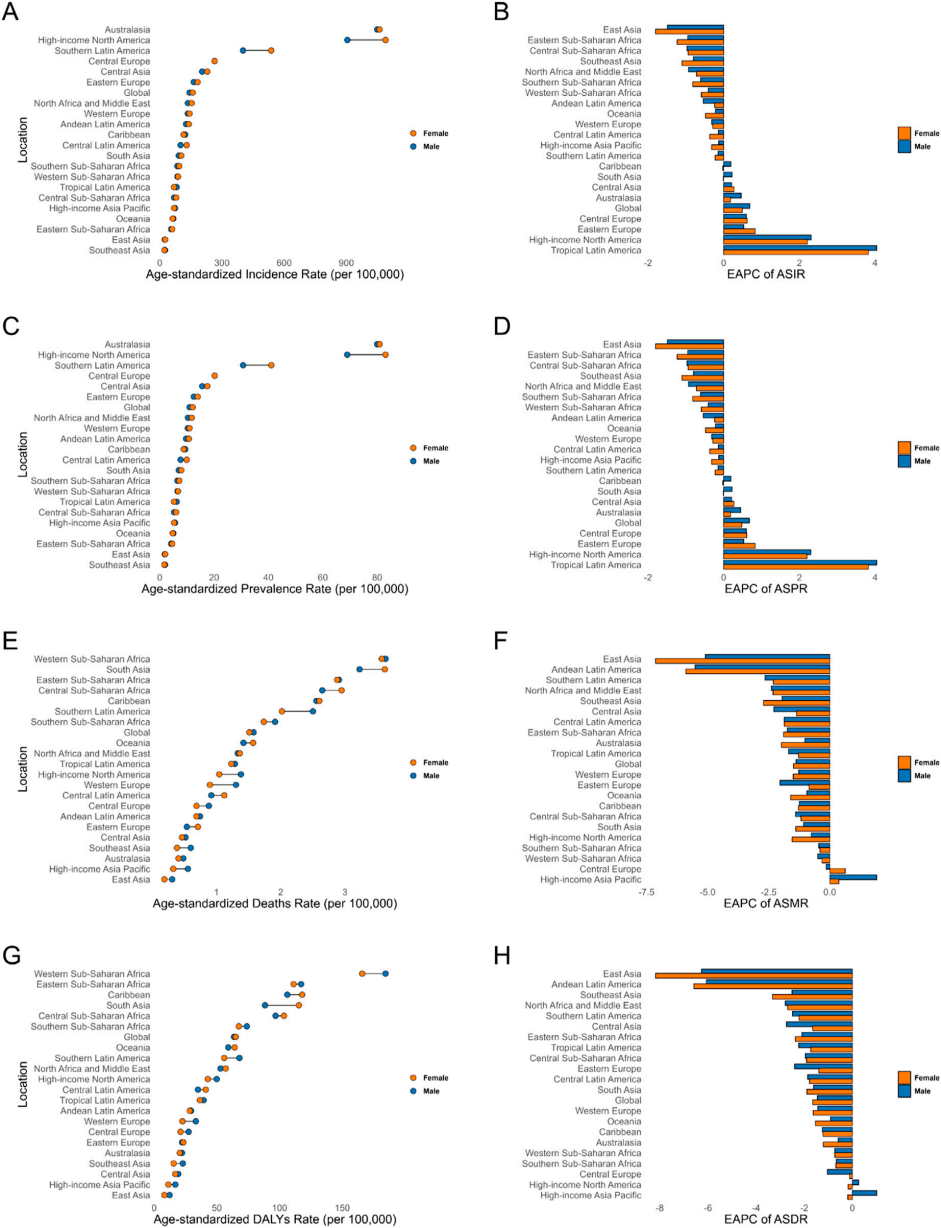
Age-Standardized Rates of Incidence, Prevalence, Deaths, and DALYs, Along with Their Estimated Annual Percentage Change (EAPC) from 1990 to 2021, in Adverse effects of medical treatment Across 21 Regions. **(A,B)** Incidence. **(C,D)** Prevalence. **(E,F)** Deaths. **(G,H)** DALYs. DALYs: Disability-Adjusted Life Years.

**FIGURE 4:**
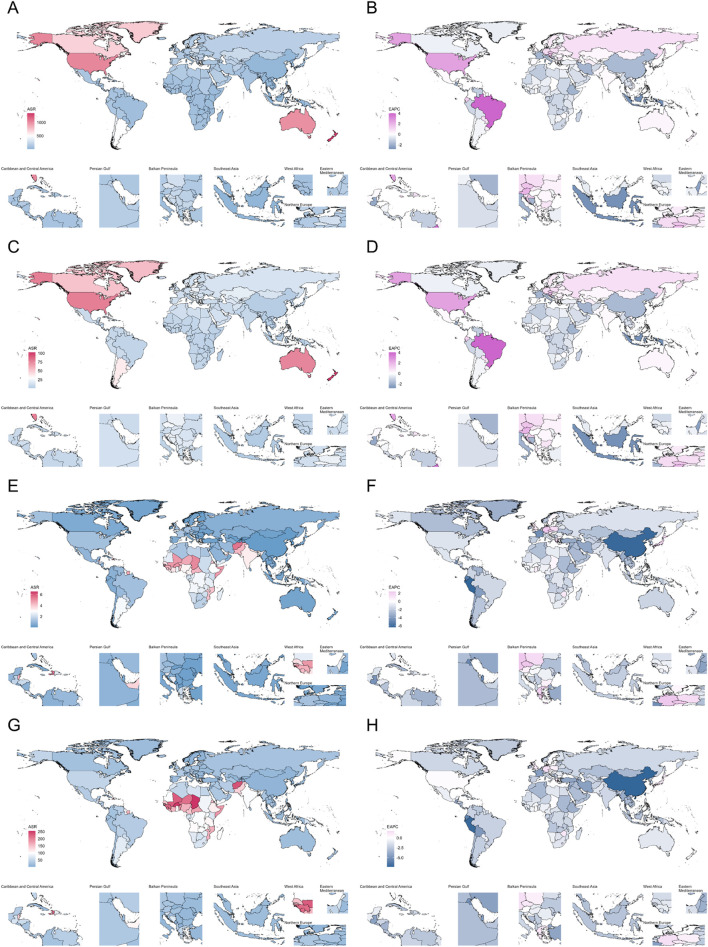
Age-Standardized Rates of Incidence, Prevalence, Deaths, and DALYs, Along with Their Estimated Annual Percentage Change (EAPC) from 1990 to 2021, in Adverse effects of medical treatment Across countries. **(A,B)** Incidence. **(C,D)** Prevalence. **(E,F)** Deaths. **(G,H)** DALYs. DALYs Disability-Adjusted Life Years.

In 2021, there were large differences in the age-standardized incidence rates (ASIR) of AEMT in 21 regions of the world. Australasia led with 1,049.66 cases per 100,000 population (95% UI: 919.10-1208.42), followed by High-income North America (993.04; 95% UI: 855.21-1146.71) and Southern Latin America (470.95; 95% UI: 415.58- 534.94). Tropical Latin America exhibited the highest EAPC (3.93; 95% CI: 2.40, 5.48) for ASIR, while East Asia had the most pronounced decrease in incidence, with an EAPC of −1.66 (95% CI: 1.85, −1.46) (Figures 3A,B, 4A,B; Table 1).

**TABLE 1:** Incidence and Prevalence of Adverse effects of medical treatment and Their Estimated Annual Percentage Change (EAPC) from 1990 to 2021 Globally, Across Different SDI Regions, and in 21 Regions.

Location	Incidence					Prevalence				
	Number		ASR per 100,000			Number		ASR per 100,000		
	1990	2021	1990	2021	Estimated Annual Percentage Change (95% CI)	1990	2021	1990	2021	Estimated Annual Percentage Change (95% CI)
Global	7407310 (6414513,8635036)	12481276 (10886793,14290630)	158.83 (138.05,183.62)	150.44 (131.19,171.81)	0.57 (0.29, 0.86)	565618 (438028,701012)	952440 (730685,1180519)	12.12 (9.35,15.07)	11.48 (8.86,14.13)	0.57 (0.29, 0.85)
High SDI	3558605 (3067811,4164732)	6708356 (5713077,7778474)	360.44 (312.04,417.69)	435.86 (377.41,501.54)	1.72 (1.32, 2.12)	271525 (207375,343158)	511387 (387625,644680)	27.51 (21.11,34.35)	33.25 (25.54,41.37)	1.72 (1.32, 2.12)
High-middle SDI	1327687 (1139860,1575956)	1598932 (1387419,1859334)	125.88 (107.8,148.52)	106.54 (92.19,124.83)	−0.47 (-0.55, -0.39)	101423 (77871,128031)	122110 (93729,153115)	9.62 (7.35,12.09)	8.14 (6.27,10.13)	−0.47 (-0.55, -0.39)
Low SDI	412756 (353365,482264)	774807 (657838,910353)	96.1 (83.13,111.56)	82.76 (72.16,95.36)	−0.49 (-0.52, -0.46)	31546 (24323,39365)	59211 (46214,73515)	7.34 (5.66,9.13)	6.32 (4.9,7.78)	−0.49 (-0.52, -0.46)
Low-middle SDI	961495 (815742,1144561)	1688772 (1442405,1993979)	95.01 (81.52,111.03)	92.56 (79.97,107.93)	0.01 (-0.04, 0.07)	73479 (56791,91498)	129043 (100777,160818)	7.26 (5.63,9.06)	7.07 (5.52,8.81)	0.01 (-0.04, 0.07)
Middle SDI	1138848 (945253,1370064)	1700233 (1445266,2020387)	69.51 (59.42,82.85)	66.93 (56.68,80.05)	−0.02 (-0.13, 0.1)	87041 (66503,109992)	129910 (100403,163165)	5.31 (4.1,6.66)	5.11 (3.97,6.44)	−0.02 (-0.13, 0.1)
Region										
Andean Latin America	49351 (42419,58094)	87238 (75529,101913)	153.34 (134.23,175.95)	133.48 (116.22,155.39)	−0.38 (-0.42, -0.34)	3771 (2939,4695)	6665 (5188,8335)	11.72 (9.14,14.54)	10.2 (7.91,12.73)	−0.38 (-0.42, -0.34)
Australasia	206938 (178322,240150)	422095 (365375,483622)	950.89 (822.64,1103.2)	1049.66 (919.1,1208.42)	0.33 (0.23, 0.42)	15791 (11978,19652)	32175 (24593,39782)	72.57 (55.37,89.37)	80.07 (62.31,98.69)	0.32 (0.23, 0.42)
Caribbean	40857 (34826,48535)	57758 (50368,66689)	119.83 (102.8,140.25)	118.82 (103.09,137.94)	0.08 (0.03, 0.14)	3122 (2429,3908)	4413 (3470,5367)	9.16 (7.16,11.32)	9.08 (7.11,11.12)	0.08 (0.03, 0.14)
Central Asia	129439 (107458,157152)	210975 (176072,252688)	211.84 (177.01,255.19)	217.64 (183.21,259.6)	0.24 (0.14, 0.34)	9890 (7542,12687)	16118 (12157,20641)	16.18 (12.31,20.73)	16.63 (12.55,21.17)	0.24 (0.14, 0.34)
Central Europe	314860 (267522,373649)	400395 (339832,473381)	232.48 (198.9,276.1)	264.15 (227.92,310.51)	0.6 (0.5, 0.69)	24047 (18307,30473)	30566 (23000,39082)	17.76 (13.53,22.57)	20.17 (15.36,25.31)	0.6 (0.5, 0.69)
Central Latin America	199497 (166849,243006)	293454 (253083,346631)	136.56 (118.03,161.43)	115.61 (99.76,137.09)	−0.26 (-0.35, -0.17)	15245 (11790,19508)	22419 (17451,28034)	10.43 (8.01,13.03)	8.83 (6.87,11.01)	−0.26 (-0.35, -0.17)
Central Sub-Saharan Africa	47353 (40345,55605)	83941 (70459,99810)	98.42 (85.81,112.72)	73.77 (64.06,84.71)	−0.96 (-1, -0.92)	3619 (2774,4531)	6415 (4900,8130)	7.52 (5.79,9.25)	5.64 (4.37,7)	−0.96 (-1, -0.92)
East Asia	445363 (354140,548265)	359559 (291717,446640)	36.78 (29.99,44.66)	24.88 (19.75,31.26)	−1.66 (-1.85, -1.46)	34049 (25502,44161)	27479 (20416,35965)	2.81 (2.11,3.62)	1.9 (1.42,2.51)	−1.66 (-1.85, -1.46)
Eastern Europe	370562 (311696,445403)	456532 (387652,537843)	149.86 (126.54,181.59)	175.87 (150.8,206.37)	0.7 (0.6, 0.81)	28305 (21469,36411)	34863 (26560,44779)	11.45 (8.72,14.7)	13.43 (10.33,17.08)	0.7 (0.6, 0.8)
Eastern Sub-Saharan Africa	130200 (110819,153394)	202700 (170242,239455)	78.55 (67.78,91.55)	57.2 (49.48,65.93)	−1.11 (-1.16, -1.05)	9952 (7682,12535)	15491 (11949,19482)	6 (4.61,7.5)	4.37 (3.4,5.4)	−1.11 (-1.16, -1.05)
High-income Asia Pacific	130076 (109039,161009)	157734 (136017,183292)	73.67 (61.46,90.93)	71.16 (60.75,84.71)	−0.23 (-0.29, -0.17)	9939 (7601,13033)	12043 (9341,15265)	5.63 (4.31,7.32)	5.44 (4.18,6.92)	−0.23 (-0.29, -0.17)
High-income North America	2535362 (2175700,2966750)	5220916 (4418047,6146695)	788.8 (678.61,920.81)	993.04 (855.21,1146.71)	2.26 (1.71, 2.81)	193383 (147560,245919)	397866 (299478,502977)	60.18 (46.1,75.78)	75.73 (58.09,95.3)	2.25 (1.71, 2.81)
North Africa and Middle East	554708 (470093,662709)	888611 (747545,1065142)	185.4 (161,217.06)	144.11 (123.17,170.68)	−0.83 (-0.87, -0.78)	42390 (32886,53342)	67903 (52709,84982)	14.16 (11.13,17.55)	11.01 (8.58,13.64)	−0.83 (-0.87, -0.78)
Oceania	4309 (3597,5217)	8432 (7198,9868)	69.64 (60.07,81)	63.68 (54.93,73.42)	−0.35 (-0.37, -0.33)	329 (251,410)	644 (501,801)	5.32 (4.14,6.54)	4.87 (3.78,5.93)	−0.35 (-0.37, -0.33)
South Asia	919611 (776147,1084841)	1745242 (1493061,2050668)	98.19 (83.71,115.12)	97.39 (84.1,113.22)	0.11 (0.04, 0.18)	70278 (54104,87409)	133356 (103918,166524)	7.5 (5.79,9.36)	7.44 (5.77,9.28)	0.11 (0.04, 0.18)
Southeast Asia	133566 (105046,167383)	162841 (133931,198595)	29.56 (24.24,35.84)	23.51 (19.33,28.52)	−0.94 (-1.02, -0.85)	10211 (7575,13432)	12445 (9482,16082)	2.26 (1.71,2.89)	1.8 (1.37,2.29)	−0.94 (-1.02, -0.85)
Southern Latin America	257245 (226115,294251)	349111 (308777,394086)	532.89 (468.86,606.44)	470.95 (415.58,534.94)	−0.2 (-0.3, -0.09)	19644 (15262,24259)	26653 (20788,32825)	40.69 (31.75,50.2)	35.96 (28.08,44.04)	−0.2 (-0.3, -0.09)
Southern Sub-Saharan Africa	52657 (43957,63024)	67268 (57543,78956)	112.53 (96.01,131.9)	89.27 (77.28,104.26)	−0.73 (-0.89, -0.56)	4024 (3100,5056)	5140 (4012,6379)	8.6 (6.77,10.73)	6.82 (5.29,8.45)	−0.73 (-0.89, -0.56)
Tropical Latin America	35179 (28361,42837)	179909 (155572,207943)	23.63 (19.42,28.07)	74.59 (64.29,86.97)	3.93 (2.4, 5.48)	2689 (2022,3449)	13745 (10670,17264)	1.81 (1.38,2.29)	5.7 (4.44,7.11)	3.93 (2.4, 5.48)
Western Europe	678782 (580901,793445)	770590 (671239,894121)	155.36 (132.65,182.35)	138.96 (120.35,161.97)	−0.3 (-0.36, -0.24)	51839 (39713,65631)	58835 (45218,74227)	11.87 (9.04,14.98)	10.61 (8.14,13.21)	−0.3 (-0.36, -0.24)
Western Sub-Saharan Africa	171395 (145450,204296)	355977 (301650,421354)	100.22 (87.03,116.46)	86.56 (75.56,99.13)	−0.49 (-0.55, -0.44)	13099 (10142,16472)	27204 (21063,34311)	7.66 (5.91,9.49)	6.61 (5.14,8.15)	−0.49 (-0.55, -0.44)

SDI, Socio-Demographic Index; EAPC, estimated annual percentage change; ASR, Age-Standardized Rate; UI, uncertainty interval; CI, confidence interval.

AEMT’s age-standardized prevalence rates (ASPR) and ASIR trends were relatively similar. ASPR in Australasia, High-income North America and Southern Latin America ranked the top three, while ASPR in Southeast Asia, East Asia and Eastern Sub-Saharan Africa ranked the last three. The increase trend of ASPR in Tropical Latin America was the most obvious, with an EAPC of 3.93 (95% CI: 2.4, 5.48), while the prevalence in East Asia decreased the most, with an EAPC of −1.66 (95% CI: 1.85, −1.46) (Figures 3C,D, 4C,D; Table 1).

In terms of mortality, Western Sub-Saharan Africa, South Asia and Eastern Sub-Saharan Africa ranked the top three, with the age-standardized mortality rates (ASMR) of 3.62 (95% UI: 2.29, 4.64), 3.41 (95% UI: 2.79,3.90) and 2.90 (95% UI: 1.87,7.12), respectively. ASMR declined over time in the vast majority of regions, except for High-income Asia-Pacific and central Europe, with EAPC of 1.19 (95% CI: 0.74, 1.64) and 0.21 (95% CI: 0.07, 0.34), respectively. The East Asia region had the most significant decrease in mortality, with an EAPC of −6.06 (95% CI: 6.33, −5.79) for ASMR (Figures 3E,F, 4E,F; Table 2).

**TABLE 2:** Deaths and DALYs of Adverse effects of medical treatment and Their Estimated Annual Percentage Change (EAPC) from 1990 to 2021 Globally, Across Different SDI Regions, and in 21 Regions.

Location	Death					DALYs				
	Number		ASR per 100,000			Number		ASR per 100,000		
	1990	2021	1990	2021	Estimated Annual Percentage Change (95% CI)	1990	2021	1990	2021	Estimated Annual Percentage Change (95% CI)
Global	108540 (94489,127769)	122330 (103910,133911)	2.4 (2.11,2.84)	1.53 (1.29,1.68)	−1.43 (-1.53, -1.34)	5744998 (4880764,6613203)	4846981 (3914845,5494171)	106.49 (91.17,122.52)	64.19 (51.06,73.11)	−1.55 (-1.59, -1.5)
High SDI	13915 (12901,14461)	19295 (17287,20486)	1.33 (1.23,1.38)	0.95 (0.87,0.99)	−1.26 (-1.69, -0.82)	384204 (364792,406072)	481279 (443819,522007)	40.17 (38.15,42.38)	31.27 (29.16,33.68)	−0.71 (-0.97, -0.44)
High-middle SDI	12994 (11479,14211)	10526 (9616,11338)	1.41 (1.26,1.54)	0.59 (0.54,0.64)	−2.92 (-3.1, -2.73)	562617 (469020,626072)	281467 (262721,306562)	57.26 (47.72,63.67)	18.61 (17.38,20.43)	−3.87 (-4.05, -3.7)
Low SDI	22818 (17768,31741)	28897 (20978,41377)	5.31 (4.01,9.21)	3.71 (2.9,5.68)	−1.07 (-1.15, -0.99)	1536300 (1169698,1958015)	1734038 (1173218,2347315)	242.8 (187.78,346.82)	150.37 (109.08,215.24)	−1.42 (-1.5, -1.35)
Low-middle SDI	34499 (29257,41873)	41801 (34407,47139)	4.22 (3.51,5.48)	2.89 (2.38,3.25)	−1.1 (-1.16, -1.04)	1864406 (1595443,2142836)	1596435 (1295388,1808680)	162.25 (137.84,194.83)	93.96 (76.55,106.1)	−1.61 (-1.67, -1.55)
Middle SDI	24227 (19319,27445)	21710 (18729,24323)	1.84 (1.49,2.12)	0.89 (0.77,1)	−2.41 (-2.52, -2.3)	1393071 (1085701,1570330)	749994 (651395,841662)	82.3 (64.9,92.74)	31.43 (27.22,35.41)	−3.15 (-3.27, -3.04)
Region										
Andean Latin America	1134 (781,1366)	427 (336,605)	3.31 (2.31,3.99)	0.71 (0.56,1.01)	−5.7 (-6.48, -4.91)	76079 (50786,92743)	18062 (14232,25238)	169.82 (116.26,204.79)	28.79 (22.68,40.34)	−6.34 (-7.06, -5.61)
Australasia	214 (198,226)	245 (212,266)	0.96 (0.88,1.01)	0.44 (0.39,0.48)	−1.55 (-2.32, -0.76)	7367 (6518,8555)	8951 (7259,11247)	34.1 (30.19,39.39)	21.22 (17.05,26.57)	−0.91 (-1.39, -0.43)
Caribbean	1263 (1113,1441)	1276 (1092,1497)	4.2 (3.75,4.69)	2.57 (2.16,3.04)	−1.27 (-1.8, -0.74)	67102 (54793,81182)	50612 (39880,63351)	184.38 (155.73,219.47)	111.91 (85.58,142.9)	−1.24 (-1.68, -0.8)
Central Asia	420 (382,456)	376 (328,422)	0.76 (0.68,0.83)	0.49 (0.43,0.54)	−1.85 (-2.19, -1.5)	22244 (20272,24484)	16374 (14353,18694)	31.47 (28.98,33.97)	17.96 (15.79,20.41)	−2.25 (-2.53, -1.97)
Central Europe	973 (926,1056)	1622 (1478,1753)	0.74 (0.71,0.8)	0.78 (0.71,0.84)	0.21 (0.07, 0.34)	37227 (34879,40188)	39948 (36840,43515)	29.66 (27.8,31.93)	24.13 (22.24,26.22)	−0.67 (-0.77, -0.56)
Central Latin America	2211 (2139,2281)	2470 (2181,2782)	1.98 (1.9,2.04)	1.03 (0.91,1.16)	−1.85 (-2.3, -1.39)	119342 (114341,124693)	90488 (78895,104210)	74.5 (72.06,77.11)	38.21 (32.96,44.32)	−1.81 (-2.17, -1.45)
Central Sub-Saharan Africa	1954 (1451,2516)	2214 (1535,3833)	4.26 (2.96,7.84)	2.81 (1.95,5.55)	−1.27 (-1.35, -1.18)	135148 (94616,177513)	123932 (83275,189575)	191.32 (143.34,267.84)	100.5 (70.26,176.12)	−1.92 (-2.05, -1.8)
East Asia	13968 (9051,16735)	4127 (3341,5563)	1.36 (0.89,1.64)	0.24 (0.2,0.32)	−6.06 (-6.33, -5.79)	889705 (560563,1080884)	142071 (118590,188601)	77.53 (48.88,94.16)	10.21 (8.68,13.15)	−7.14 (-7.44, -6.83)
Eastern Europe	2250 (2121,2412)	2094 (1928,2290)	0.9 (0.85,0.96)	0.67 (0.62,0.72)	−1.31 (-1.53, -1.09)	85153 (80603,90519)	60940 (56652,65844)	37.23 (35.36,39.17)	23.38 (21.8,25.11)	−1.88 (-2.19, -1.57)
Eastern Sub-Saharan Africa	8127 (5593,14104)	8001 (5380,16998)	4.97 (3.08,12.64)	2.9 (1.87,7.12)	−1.82 (-1.86, -1.77)	566600 (397046,789080)	483338 (323740,898527)	230.32 (155.01,443.26)	114.33 (76.83,244.4)	−2.24 (-2.28, -2.2)
High-income Asia Pacific	650 (600,721)	1946 (1667,2136)	0.37 (0.34,0.41)	0.43 (0.39,0.46)	1.19 (0.74, 1.64)	22604 (20998,26059)	38289 (34819,40976)	13.84 (12.76,16.28)	13.94 (13.07,14.78)	0.46 (0.15, 0.78)
High-income North America	4877 (4494,5073)	7254 (6584,7615)	1.4 (1.3,1.46)	1.19 (1.1,1.25)	−1.18 (-1.71, -0.64)	144671 (133595,158317)	227720 (205270,256543)	46.08 (42.74,50.45)	46.06 (41.92,51.52)	0.04 (-0.18, 0.26)
North Africa and Middle East	7619 (5907,9598)	6888 (5509,8122)	2.84 (2.18,3.89)	1.35 (1.08,1.56)	−2.36 (-2.38, -2.33)	460503 (352921,561198)	323833 (257299,387329)	131.01 (101.18,162.55)	54.92 (43.72,65.43)	−2.74 (-2.78, -2.7)
Oceania	99 (58,211)	152 (93,304)	2.21 (1.3,4.77)	1.49 (0.89,3.05)	−1.3 (-1.46, -1.14)	5741 (3550,11394)	8443 (5392,15603)	90.18 (53.42,192.21)	61.49 (38.16,119.99)	−1.25 (-1.39, -1.11)
South Asia	35956 (30209,44432)	48186 (39550,55133)	5.11 (4.22,6.65)	3.41 (2.79,3.9)	−1.24 (-1.32, -1.16)	1790302 (1488089,2092969)	1634022 (1348111,1853129)	179.55 (151.53,219.1)	101.55 (83.54,115.67)	−1.76 (-1.82, -1.7)
Southeast Asia	3585 (2806,6072)	3035 (2432,5026)	0.96 (0.75,1.55)	0.49 (0.39,0.8)	−2.3 (-2.35, -2.25)	216700 (160989,388379)	125297 (98269,216052)	45.33 (35.38,78.69)	19.07 (14.94,33.53)	−2.87 (-2.93, -2.81)
Southern Latin America	2531 (2409,2633)	1944 (1785,2071)	5.87 (5.53,6.13)	2.24 (2.07,2.38)	−2.47 (-3.15, -1.78)	70897 (68708,73249)	48256 (45265,51117)	151.82 (146.85,156.98)	61.46 (58.04,65.24)	−2.35 (-2.95, -1.75)
Southern Sub-Saharan Africa	879 (724,1055)	1145 (914,1296)	2.22 (1.76,2.82)	1.82 (1.45,2.04)	−0.44 (-0.71, -0.16)	50104 (42503,57357)	52217 (41167,60431)	96.12 (80.1,114.45)	70.37 (55.5,80.82)	−0.69 (-0.95, -0.42)
Tropical Latin America	1738 (1663,1813)	3095 (2866,3267)	1.72 (1.62,1.79)	1.26 (1.16,1.33)	−1.48 (-1.82, -1.13)	78573 (73059,83488)	90474 (85572,95035)	60.08 (56.6,63.14)	37.91 (35.66,40.13)	−2 (-2.35, -1.64)
Western Europe	8796 (8153,9142)	10740 (9419,11489)	1.6 (1.49,1.66)	1.08 (0.98,1.14)	−1.37 (-1.94, -0.79)	202544 (194619,208415)	201457 (184810,212624)	43.13 (41.76,44.31)	27.62 (26.05,28.99)	−1.51 (-2.07, -0.94)
Western Sub-Saharan Africa	9296 (7422,11781)	15095 (8799,18823)	4.4 (3.53,5.93)	3.62 (2.29,4.64)	−0.39 (-0.57, -0.22)	696391 (538135,886635)	1062255 (598466,1352845)	241.31 (193.58,305.57)	175.48 (103.3,219.03)	−0.72 (-0.9, -0.55)

SDI, Socio-Demographic Index; EAPC, estimated annual percentage change; DALYs, Disability-Adjusted Life Years; ASR, Age-Standardized Rate; UI, uncertainty interval; CI, confidence interval.

The top three DALYs rates were in Western Sub-Saharan Africa, Eastern Sub-Saharan Africa and Caribbean, with DALYs rates of 175.48 (95% UI: 103.3,219.03), 114.33 (95% UI: 76.83,244.4) and 111.91 (95% UI: 85.58,142.9). Age-standardized DALYs rates (ASDR) declined over time in the vast majority of regions, except for the High-income Asia-Pacific and High-income North America male cohorts, where total EAPC was 0.46 (95% CI: 0.15, 0.78) and 0.04 (95% CI: -0.18, 0.26) for both regions. The most significant decrease in DALYs rate was seen in East Asia, where the EAPC for ASDR was −7.14 (95% CI: -7.44, −6.83) (Figures 3G,H, 4G,H; Table 2).

### 3.3 Age- and sex-specific patterns of AEMT

**FIGURE 5:**
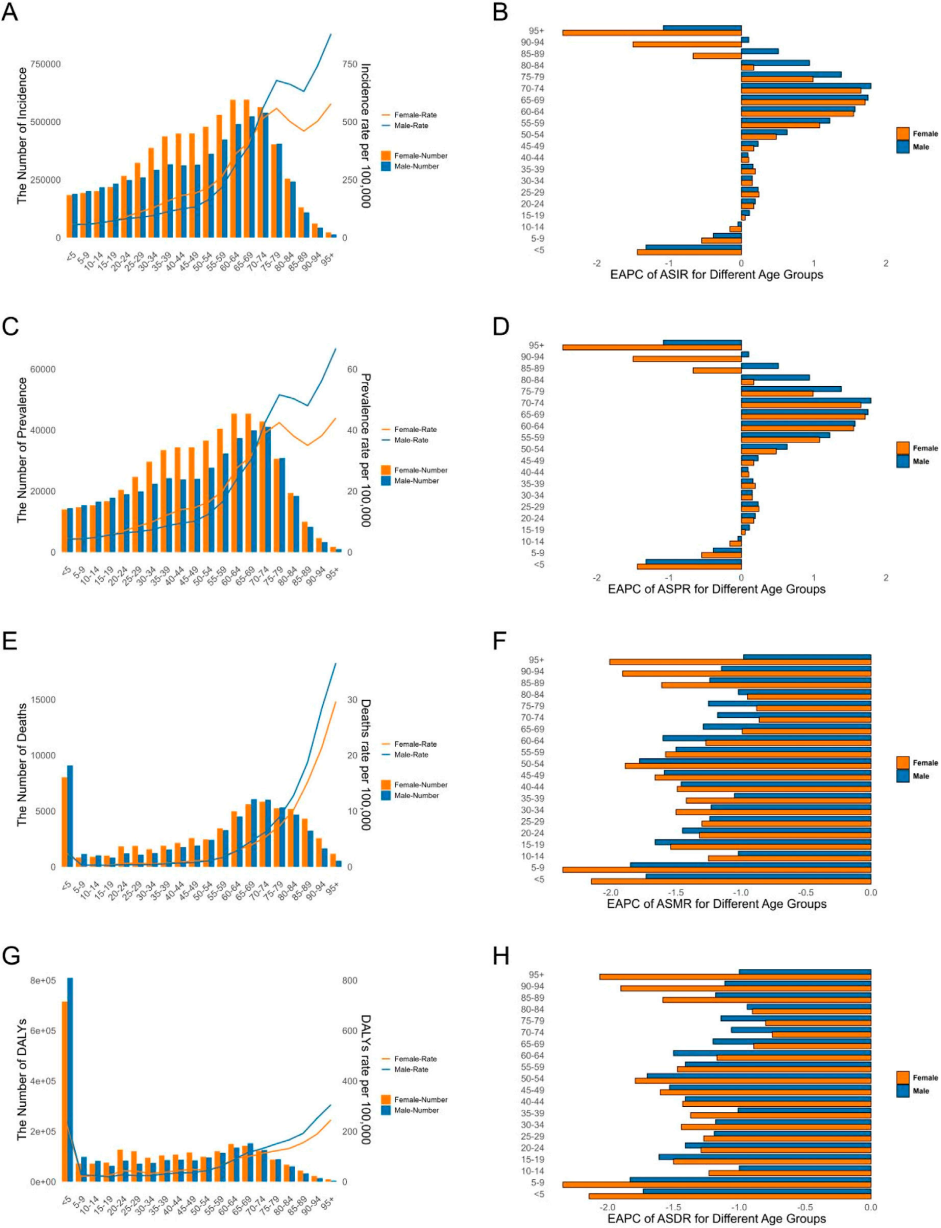
Age-Standardized Rates of Incidence, Prevalence, Deaths, and DALYs for Adverse effects of medical treatment in 2021, Including Their Estimated Annual Percentage Change (EAPC) from 1990 to 2021, for Different Age Groups. **(A,B)** Incidence. **(C,D)** Prevalence. **(E,F)** Deaths. **(G,H)** DALYs. DALYs Disability-Adjusted Life Years.

Significant differences were observed in the incidence of AEMT in different age and gender groups. The highest incidence of AEMT in both men and women was in those aged 95 years or older. The highest incidence number for males were in the 70-74 age group, while that for females were in the 65-69 age group, with a total incidence number of 1119328 (95% UI: 755592,1649253). From 1990 to 2021, there was a significant decline in the incidence rate for those under 14 years of age, elderly females over 85 years of age, and elderly males over 95 years of age, with increasing trends in all other age groups. The most pronounced decline was seen in the population aged 95 years and older, for which the EAPC for incidence rate was −2.03 (95% CI: -2.34, −1.72) (Figures 5A,B; Supplementary Table S1).

In 2021, the highest prevalence number of AEMT was in men aged 70-74 years and in women aged 65-69 years. In terms of prevalence rate, the highest prevalence rate was in men and women over 95 years of age. Similar to incidence, there was a significant decline in prevalence in the under 14 years old, elderly females over 85 years of age, and elderly males over 95 years of age, with varying degrees of increasing prevalence in all other age groups. The most pronounced decline was seen in the population over 95 years of age, where the EAPC for prevalence rate was −2.03 (95% CI: -2.34, −1.72) (Figures 5C,D; Supplementary Table S1).

Infants and children younger than 5 years of age had the highest number of AEMT deaths among all age groups, with a total of 17,141 (95% UI: 11096,21840). The mortality rate of AEMT among those older than 5 years of age continued to increase with age, with the highest mortality rate among those 95 years of age and older, at 31.56 per 100,000 (95% UI: 23.69,35.88). Mortality rates declined in all age groups, with the most pronounced decline in the 5-9 years old group, which had an EAPC for mortality of −2.08 (95% CI: 2.22, −1.94) (Figures 5E,F; Supplementary Table S2).

Infants and children younger than 5 years of age had the highest number of DALYs for AEMT at 1,525,817 (95% UI: 992301,1940861). The rate of DALYs increased with age for those older than 5 years, with the highest rate of 262.06 (95% UI: 198.62,297.64) per 100,000 for those older than 95 years. The rate of DALYs declined in all age groups, with the most pronounced decline in the 5-9 years old group, which had an EAPC of −2.05 (95% CI: 2.19, −1.92) (Figures 5G,H; Supplementary Table S2).

### 3.4 Regional and national burden of AEMT by SDI

**FIGURE 6:**
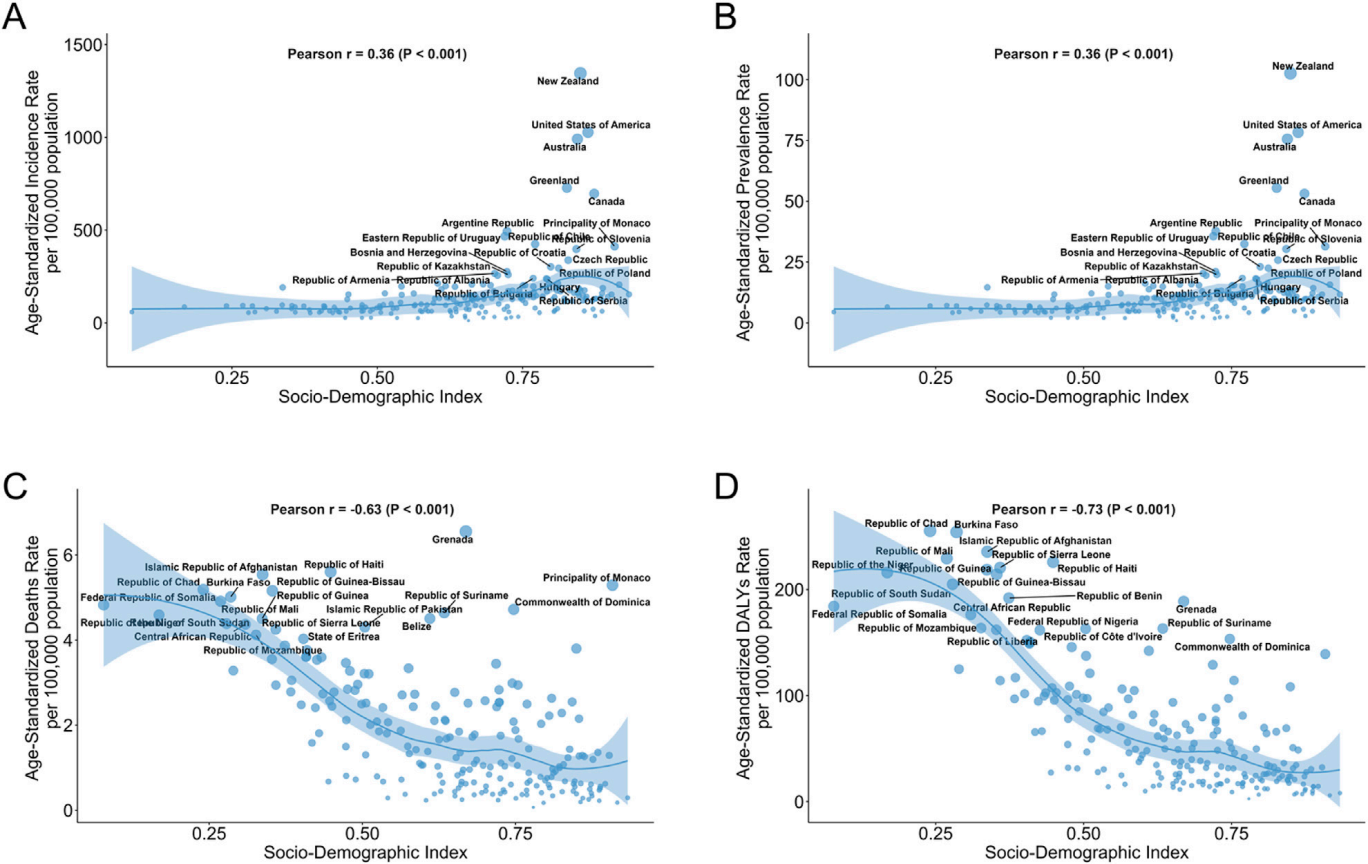
Burden of Adverse effects of medical treatment Across 204 countries by SDI. **(A)** ASIR for 204 countries by SDI. **(B)** ASPR for 204 countries by SDI. **(C)** ASMR for 204 countries by SDI. **(D)** ASDR for 204 countries by SDI. The chart highlights the 15 countries with the heaviest disease burden. ASIR Age-Standardized Incidence Rate, ASPR Age-Standardized Prevalence Rate, ASMR Age-Standardized Mortality Rate, ASDR Age-Standardized Disability-Adjusted Life Year Rate, SDI Socio-Demographic Index.

In the 21 regions, overall ASIR (Pearson r = 0.50, p < 0.001) and ASPR (Pearson r = 0.50, p < 0.001) showed a positive correlation with SDI, while ASDR (Pearson r = −0.74, p < 0.001) and ASMR (Pearson r = −0.80, p < 0.001) showed a negative correlation with SDI (Supplementary Figure S1). Consistent with the overall trend, High-income North America and Australasia regions led in SDI values, ASIR and ASPR, while ASDR and ASMR were lower. Interestingly, East Asia region exhibited the middle level of SDI, but ASIR, ASPR, ASDR and ASMR were at the lowest. In addition, High-income Asia Pacific region exhibited very high SDI, but ASIR, ASPR, ASDR and ASMR were relatively low (Supplementary Figure S1). Out of 204 countries, New Zealand, United States of America and Australia, the three rich countries, were in the top 3 for ASIR and ASPR (Figures 6A,B). Grenada was in the top position for ASDR, while Republic of Chad had the top ASMR (Figures 6C,D).

### 3.5 Health inequality analysis

As shown by the health inequality analysis, the SII of ASIR increased from 91.410 (95% CI: 64.967, 117.853) in 1990 to 98.728 (95% CI: 77.706, 119.750) in 2021. Similarly, the ASPR increased to 7.542 (95% CI: 5.936, 9.149). The ASDR and ASMR decreased to −2.396 (95% CI: -2.876, −1.916) and −101.395 (95% CI: -116.741, −86.049) in 2021.

**FIGURE 7:**
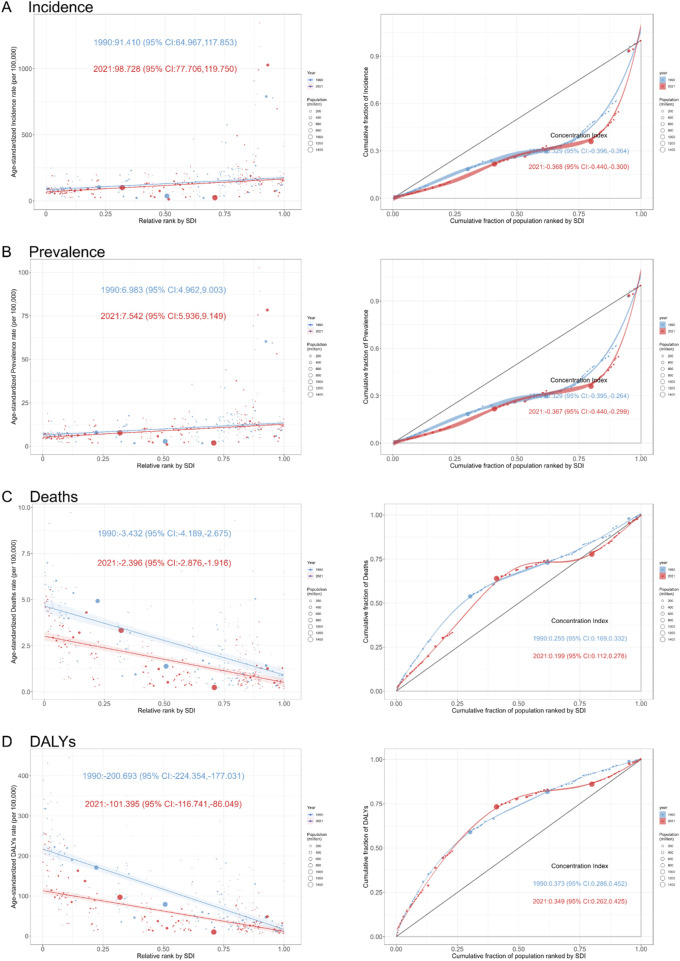
Slope Index of Inequality (Left) and Concentration Index (Right) Curves for Adverse effects of medical treatment in 1990 and 2021. **(A)** Incidence. **(B)** Prevalence. **(C)** Deaths. **(D)** DALYs. DALYs: Disability-Adjusted Life Years.

On the other hand, the CI of ASIR decreased from −0.329 (95% CI: -0.396, −0.264) in 1990 to −0.368 (95% CI: -0.440, −0.300) in 2021, while the CI of ASDR decreased from −0.329 (95% CI: 0.395, −0.264) in 1990 to −0.367 (95% CI: 0.440, −0.299) in 2021. The ASDR and ASMR decreased to 0.199 (95% CI: 0.112, 0.278) and 0.349 (95% CI: 0.260, 0.425) in 2021 (Figure 7; Supplementary Table S3).

### 3.6 Forecasting the future trend of AEMT

**FIGURE 8:**
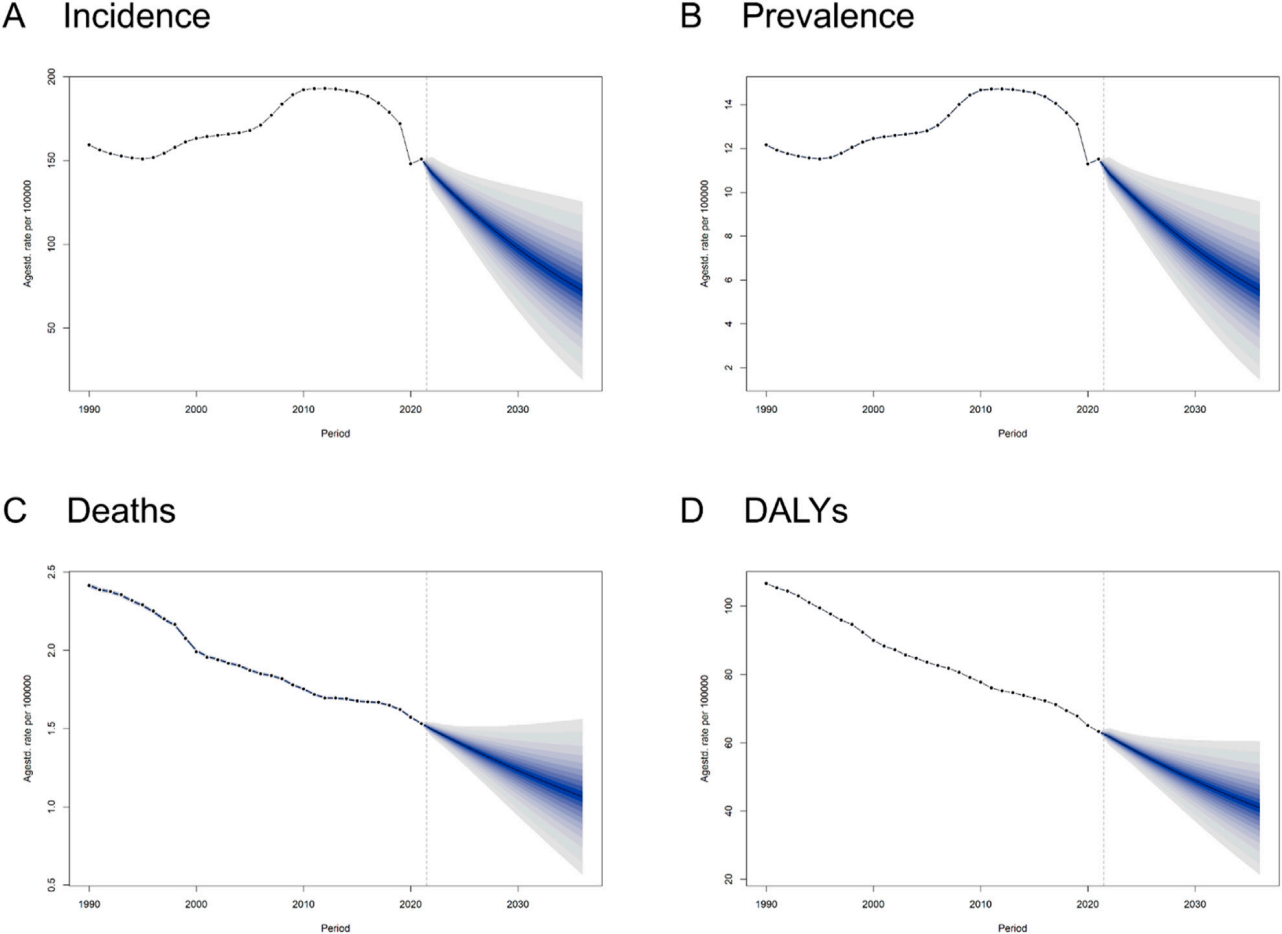
Trends and Forecasts of the Burden of Adverse effects of medical treatment Globally Until 2036. **(A)** Incidence. **(B)** Prevalence. **(C)** Deaths. **(D)** DALYs Disability-Adjusted Life Years.

As predicted by the BAPC model, the incidence number of AEMT by 2036 would decrease to 6493037 (95% UI: 1741113,11244960), with the ASIR to 72.33 (95% UI: 19.4,125.27) per 100000 population. Similarly, the ASPR, ASDR and ASMR would drop to 5.53 (95% UI: 1.47,9.58), 1.07 (95% UI: 0.57,1.56) and 40.98 (95% UI: 21.52,60.43) per 100000 population by 2036, respectively (Figure 8; Supplementary Table S4).

## 4 Discussion

Currently, the estimates regarding the global burden of AEMT remain based on the GBD 2019 data (Kong et al., 2024; Lin, 2025), which revealed a significant downward trend in global age-standardized mortality rates and DALYs rates. Accordingly, we analyzed the 2021 GBD data to update the global incidence, prevalence, mortality and DALYs of AEMT. Temporal trends showed a decrease in incidence and prevalence in 2020, followed by an increase in 2021, likely reflecting the impact of the COVID-19 pandemic on healthcare utilization and reporting. The continuous increase in AEMT incidence and prevalence may reflect greater awareness across regions, highlighting the need for intensified prevention efforts. Regarding sex, women had the highest number of AEMT cases in 2016, followed by a decline, whereas men reached their peak in 2017. The numerical value of DALYs was relatively stable, with only small differences between men and women. This suggests that the medical system may be effectively controlling the risk of AEMT, or there may be underreporting in event data (Khan et al., 2020). This issue still requires further investigation, so that we can make a more informed assessment.

In 2021, there were significant differences in adverse events due to medical treatment (AEMT) among countries and regions worldwide. Australia had the highest ASIR, followed by high-income North America and southern Latin America. A similar trend was observed in the ASPR. This pattern likely reflects the greater emphasis that hospital authorities in high-income countries place on detecting and reporting AEMT (Khan et al., 2020). The mortality rate of AEMT had been declining annually in most regions. Notably, in the Asia-Pacific and Central Europe regions among high-income countries, the EAPC were 1.19 and 0.21 respectively. In East Asia, this trend declined most significantly, with an EAPC of −6.06. This suggests that there were valuable aspects to learn from East Asia’s handling of AEMT. However, the specific reasons behind the decline in this trend still require further investigation (Kong et al., 2024).

Among regions with the highest burden of disease as measured by DALYs, the top three were Western Sub-Saharan Africa, Eastern Sub-Saharan Africa, and the Caribbean. This may be related to the scarcity of medical resources and limited access to highly trained healthcare professionals in these areas (Woode Owusu et al., 2023; Phadungsombat et al., 2022). Furthermore, the Caribbean experienced a severe outflow of doctors, which, combined with frequent natural disasters, further exacerbated the local shortage of medical resources. ASDR had been declining over time in most regions; however, ASDR in certain parts of the Asia-Pacific and North America had been increasing significantly. However, there was an imbalance in the health status of these two regions (Hume, 2010; Steenhoff et al., 2019). Therefore, the main factors leading to the increase in the ASDR still need to be explored further. Addressing these factors is essential to reduce the ASDR and maintain the health of the people in the regions.

We found that AEMT prevalence rates vary across age groups. In 2021, the age group with the highest number of male AEMT patients was 70–74 years, while for female patients, it was 65–69 years. This indicates that, under the same treatment principles, adverse medical events are more likely to occur in these age group during the medical process, reflecting biological vulnerability and the need for heightened clinical vigilance (Lin, 2025; Shin et al., 2024). In addition, it is worth noting that among infants and toddlers under the age of 5, the number of deaths due to AEMT was the highest compared to other age groups (Virgo et al., 2020). The total cases reached 17,141. Subsequently, as age increases, the mortality rate of AEMT also increases. This trend correlates with increased susceptibility to diseases as age advances. However, AEMT among infants and young children are more likely to result in serious consequences.

Interestingly, conventional perception holds that countries with high SDI have more medical resources and thus fewer AEMT cases. Yet, our study found that countries with high SDI, such as New Zealand, the United States, and Australia, had higher ASIR and ASPR of AEMT, possibly due to better detection rates. Despite higher incidence in high SDI regions, ASDR and ASMR remain lower, suggesting effective healthcare management. Therefore, the healthcare system in low- or middle-SDI regions need further strengthening. Especially in Grenada, the age-standardized mortality rate of AEMT ranked highest, suggesting that the country should focus on improving medical care (Ammar et al., 2023; Schnaubelt et al., 2023).

Based on the results of health inequality analysis, we found that the absolute and relative inequalities in incidence and prevalence rates were expanding, and health advantages were concentrated in low SDI regions. The mortality and DALYs rate showed completely opposite trends. The inequality had gradually decreased, and the health advantages were concentrated in high SDI regions. Our study results have significant implications for clinical practice and health policy formulation. Firstly, in low SDI regions, the detection and treatment of AEMT require urgent improvement. These regions may face issues such as insufficient resources and ineffective supervision in their healthcare systems (Oke and Sibomana, 2025). Secondly, prevention and control strategies for AEMT should be included in policy discussions, with the aim of playing a positive role in improving patient outcomes and reducing medical risks. These findings emphasize the importance of comprehensively assessing inequalities within the healthcare system and suggest that policymakers should consider the specific needs of different SDI regions when allocating resources and implementing medical interventions, in order to achieve a more equitable healthcare system (Shah et al., 2019; Costa-Font et al., 2024; Edlin and Yadav, 2022).

### 4.1 Limitations

Although this study comprehensively quantified the burden of AEMT at the global, regional and national levels using GBD 2021 data, there are still several inherent limitations. Firstly, the data integration for GBD 2021 relies on an extremely wide but highly heterogeneous set of global data sources. There are significant differences in the monitoring, identification, recording, and coding of AEMT among different countries and regions. Secondly, the underreporting and misreporting of AEMT (especially drug-related incidents) is a global problem. Thirdly, in areas with scarce data, GBD heavily relies on statistical modeling and uses data from neighboring countries or similar regions, which may introduce biases. Finally, there are differences in medical accessibility and intervention measures between high and low SDI regions, which may lead to an underestimation of AEMT in the low SDI regions. Therefore, the burden estimates of GBD 2021 AEMT presented in this study should be regarded as a systematic, standardized but potentially underestimated quantitative assessment of the global burden of medical adverse events, based on the best but imperfect available data and methods. These limitations highlight the urgency of investing in more powerful and standardized global drug safety monitoring and patient safety surveillance systems to obtain more accurate and detailed AEMT data.

## 5 Conclusion

This study provides the comprehensive global, regional, and national assessment of AEMT over the past three decades and offers forecasts to 2036. In 2021, there were 12,481,276 new AEMT cases, 122,330 deaths, and 4,846,981 DALYs loss worldwide, with the highest mortality and DALY rates observed in low-SDI regions such as Western Sub-Saharan Africa. Although age-standardized mortality and DALY rates have declined globally, the rising incidence and prevalence in high-SDI regions highlight ongoing challenges. Our forecasts suggest that the global ASDR will decline to 40.98 per 100,000 person-years by 2036, yet regional disparities will persist. These findings underscore the urgent need to strengthen patient safety measures, improve medical education and training, enhance surveillance systems for early detection and reporting, and allocate resources strategically to high-burden regions. By implementing these strategies, healthcare systems worldwide can reduce preventable harm and achieve more equitable health outcomes.

## Data Availability

Publicly available datasets were analyzed in this study. This data can be found here: https://vizhub.healthdata.org/gbd-results/.
