# Supplementary material for: Global burden and trends of adverse effects of medical treatment, 1990–2021: an analysis from the global burden of disease study 2021
Source: Front Pharmacol. 2025 Sep 12;16:1655864. doi: 10.3389/fphar.2025.1655864 (PMC12464418; doi:10.3389/fphar.2025.1655864)
Supplement: Supplementary file 1 [file Supplementaryfile1.docx]

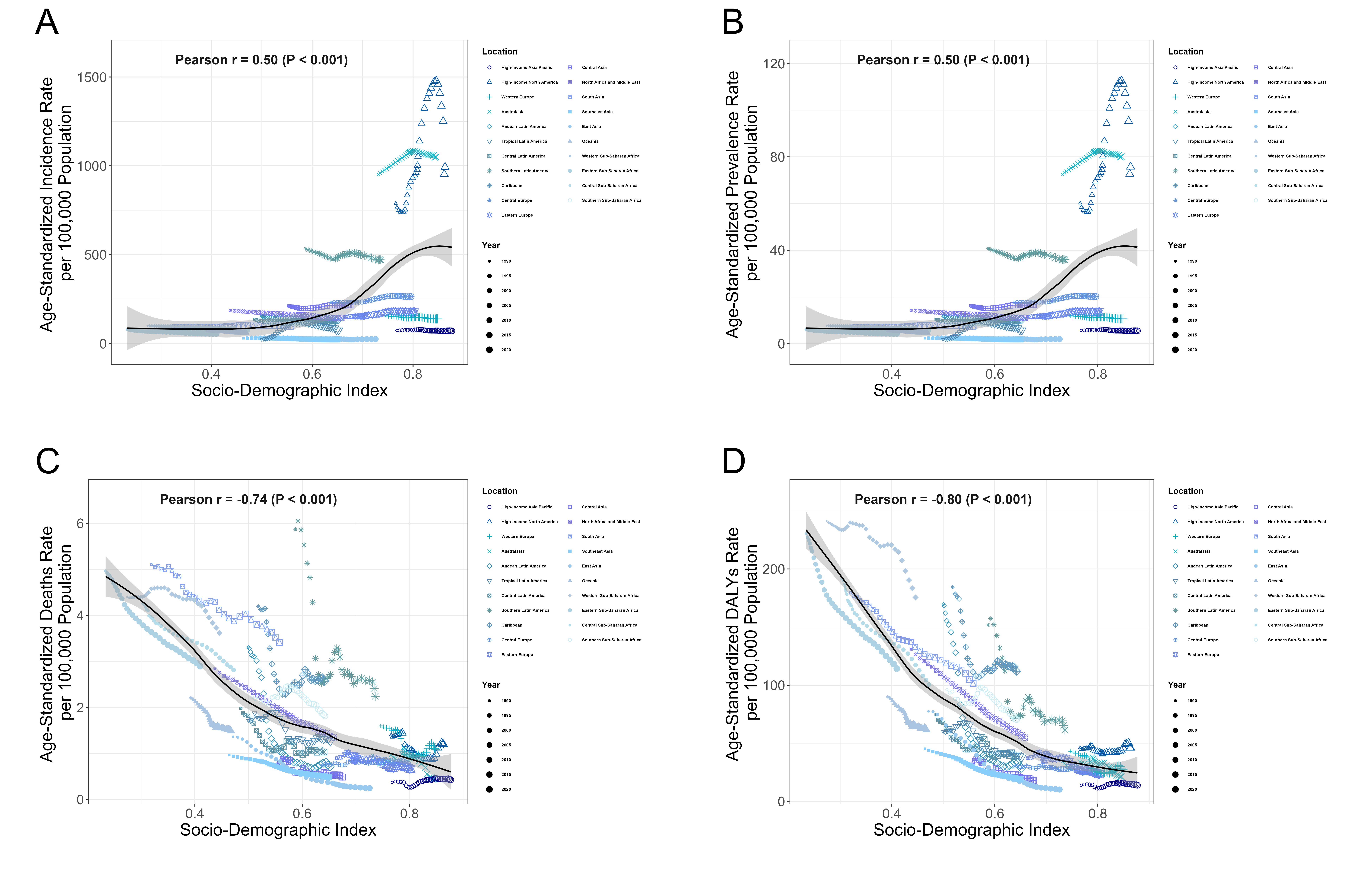


**Fig. S1** Burden of Adverse effects of medical treatment Across 21 Regions by SDI. (A) ASIR for 21 regions by SDI from 1990 to 2021. (B) ASPR for 21 regions by SDI from 1990 to 2021. (C) ASMR for 21 regions by SDI from 1990 to 2021. (D) ASDR for 21 regions by SDI from 1990 to 2021. ASIR Age-Standardized Incidence Rate, ASPR Age-Standardized Prevalence Rate, ASMR Age-Standardized Mortality Rate, ASDR Age-Standardized Disability-Adjusted Life Year Rate, SDI Socio-Demographic Index.

**Table S1 Global Incidence and prevalence of Adverse effects of medical treatment and their EAPC from 1990 to 2021 by age groups.**

| **Age Groups** | **Incidence** |  |  |  |  | **Prevalence** |  |  |  |  |
| --- | --- | --- | --- | --- | --- | --- | --- | --- | --- | --- |
|  | **Number** |  | **Rate per 100,000** |  |  | **Number** |  | **Rate per 100,000** |  |  |
|  | **1990** | **2021** | **1990** | **2021** | **Estimated Annual Percentage Change (95% CI)** | **1990** | **2021** | **1990** | **2021** | **Estimated Annual Percentage Change (95% CI)** |
| <5 | 533611 (396059,674647) | 372191 (270337,470393) | 86.07 (63.89,108.82) | 56.55 (41.07,71.47) | -1.37 (-1.48, -1.27) | 40812 (27950,54637) | 28463 (19333,38506) | 6.58 (4.51,8.81) | 4.32 (2.94,5.85) | -1.38 (-1.48, -1.27) |
| 5-9 | 396805 (238848,619537) | 394267 (238533,623160) | 68 (40.93,106.17) | 57.39 (34.72,90.7) | -0.47 (-0.55, -0.4) | 30316 (18008,48472) | 30118 (17910,48186) | 5.2 (3.09,8.31) | 4.38 (2.61,7.01) | -0.47 (-0.55, -0.4) |
| 10-14 | 359950 (215541,611137) | 418274 (251694,719376) | 67.19 (40.24,114.09) | 62.74 (37.76,107.91) | -0.1 (-0.19, -0.01) | 27475 (15513,46917) | 31926 (17880,54150) | 5.13 (2.9,8.76) | 4.79 (2.68,8.12) | -0.1 (-0.19, -0.01) |
| 15-19 | 377511 (220613,610739) | 451134 (268653,730186) | 72.68 (42.47,117.58) | 72.3 (43.05,117.02) | 0.08 (0, 0.16) | 28869 (16159,48287) | 34500 (19592,57768) | 5.56 (3.11,9.3) | 5.53 (3.14,9.26) | 0.08 (0, 0.16) |
| 20-24 | 413497 (236217,633388) | 515033 (304025,773853) | 84.03 (48,128.71) | 86.25 (50.91,129.59) | 0.18 (0.09, 0.28) | 31643 (17204,49240) | 39411 (22488,60772) | 6.43 (3.5,10.01) | 6.6 (3.77,10.18) | 0.18 (0.09, 0.28) |
| 25-29 | 445238 (239820,680648) | 583256 (322209,888084) | 100.59 (54.18,153.78) | 99.14 (54.77,150.95) | 0.23 (0.1, 0.37) | 34056 (18392,52929) | 44608 (24257,69015) | 7.69 (4.16,11.96) | 7.58 (4.12,11.73) | 0.23 (0.1, 0.37) |
| 30-34 | 469185 (274949,698036) | 680436 (409812,1003876) | 121.73 (71.34,181.11) | 112.57 (67.8,166.07) | 0.15 (-0.02, 0.33) | 35886 (20380,55087) | 52039 (30357,79188) | 9.31 (5.29,14.29) | 8.61 (5.02,13.1) | 0.15 (-0.02, 0.33) |
| 35-39 | 490219 (288362,717259) | 753913 (463640,1085767) | 139.17 (81.86,203.63) | 134.42 (82.66,193.59) | 0.18 (0.06, 0.3) | 37491 (20922,56955) | 57655 (32915,86601) | 10.64 (5.94,16.17) | 10.28 (5.87,15.44) | 0.18 (0.06, 0.3) |
| 40-44 | 459957 (271539,684857) | 760960 (458308,1119675) | 160.55 (94.78,239.06) | 152.12 (91.62,223.82) | 0.1 (-0.02, 0.23) | 35159 (19493,54648) | 58166 (32948,87977) | 12.27 (6.8,19.08) | 11.63 (6.59,17.59) | 0.1 (-0.02, 0.23) |
| 45-49 | 406063 (252853,603976) | 764491 (483795,1112439) | 174.88 (108.9,260.12) | 161.45 (102.17,234.94) | 0.21 (-0.04, 0.46) | 31012 (18502,47119) | 58385 (35058,87712) | 13.36 (7.97,20.29) | 12.33 (7.4,18.52) | 0.21 (-0.04, 0.46) |
| 50-54 | 432508 (266620,641801) | 841095 (534220,1213164) | 203.46 (125.43,301.92) | 189.04 (120.07,272.67) | 0.55 (0.17, 0.94) | 33028 (19737,51608) | 64227 (39256,97119) | 15.54 (9.28,24.28) | 14.44 (8.82,21.83) | 0.55 (0.17, 0.94) |
| 55-59 | 450258 (293693,677288) | 953940 (631728,1400202) | 243.12 (158.58,365.71) | 241.06 (159.64,353.83) | 1.15 (0.76, 1.54) | 34353 (21417,52081) | 72775 (46858,109469) | 18.55 (11.56,28.12) | 18.39 (11.84,27.66) | 1.15 (0.76, 1.54) |
| 60-64 | 497504 (327995,722593) | 1085575 (742743,1550642) | 309.76 (204.22,449.91) | 339.19 (232.07,484.5) | 1.56 (1.14, 1.99) | 37943 (23597,57291) | 82788 (53987,124395) | 23.62 (14.69,35.67) | 25.87 (16.87,38.87) | 1.56 (1.14, 1.99) |
| 65-69 | 486400 (328604,727868) | 1119328 (755592,1649253) | 393.5 (265.84,588.84) | 405.79 (273.92,597.9) | 1.73 (1.2, 2.26) | 37102 (23424,57397) | 85333 (53308,131644) | 30.02 (18.95,46.43) | 30.94 (19.33,47.72) | 1.73 (1.2, 2.26) |
| 70-74 | 422398 (270828,627945) | 1102703 (699455,1630697) | 498.93 (319.9,741.72) | 535.71 (339.81,792.22) | 1.73 (1.24, 2.22) | 32165 (19262,50615) | 83908 (51636,128303) | 37.99 (22.75,59.78) | 40.76 (25.09,62.33) | 1.72 (1.23, 2.21) |
| 75-79 | 373175 (258534,517282) | 807807 (566475,1076368) | 606.24 (420,840.35) | 612.51 (429.52,816.14) | 1.2 (0.73, 1.67) | 28411 (18188,41851) | 61447 (39446,86221) | 46.16 (29.55,67.99) | 46.59 (29.91,65.38) | 1.18 (0.72, 1.65) |
| 80-84 | 233249 (155110,322363) | 497173 (352621,677887) | 659.34 (438.46,911.25) | 567.66 (402.61,773.99) | 0.54 (-0.15, 1.22) | 17761 (11316,25971) | 37839 (24878,55630) | 50.21 (31.99,73.41) | 43.2 (28.4,63.52) | 0.53 (-0.15, 1.21) |
| 85-89 | 109277 (77521,151052) | 239893 (179676,322210) | 723.16 (513.01,999.61) | 524.68 (392.98,704.72) | -0.19 (-0.97, 0.6) | 8304 (5477,12065) | 18234 (12179,25748) | 54.95 (36.24,79.84) | 39.88 (26.64,56.32) | -0.19 (-0.97, 0.6) |
| 90-94 | 38340 (25904,54420) | 103734 (72925,143726) | 894.7 (604.51,1269.97) | 579.87 (407.65,803.41) | -0.94 (-1.56, -0.31) | 2911 (1889,4323) | 7879 (5192,11424) | 67.93 (44.08,100.88) | 44.04 (29.02,63.86) | -0.94 (-1.55, -0.31) |
| 95+ | 12165 (7116,19452) | 36073 (21223,55174) | 1194.85 (698.93,1910.62) | 661.86 (389.39,1012.31) | -2.03 (-2.34, -1.72) | 924 (512,1509) | 2741 (1579,4350) | 90.76 (50.24,148.19) | 50.28 (28.98,79.81) | -2.03 (-2.34, -1.72) |

**EAPC Estimated Annual Percentage Change, ASR Age-Standardized Rate, UI uncertainty interval, CI confidence interval.**

**Table S2 Global Death and DALYs of Adverse effects of medical treatment and their EAPC from 1990 to 2021 by age groups.**

| **Age Groups** | **Death** |  |  |  |  | **DALYs** |  |  |  |  |
| --- | --- | --- | --- | --- | --- | --- | --- | --- | --- | --- |
|  | **Number** |  | **Rate per 100,000** |  |  | **Number** |  | **Rate per 100,000** |  |  |
|  | 1990 | 2021 | 1990 | 2021 | Estimated Annual Percentage Change (95% CI) | 1990 | 2021 | 1990 | 2021 | Estimated Annual Percentage Change (95% CI) |
| <5 | 32251 (24594,39845) | 17141 (11096,21840) | 5.2 (3.97,6.43) | 2.6 (1.69,3.32) | -1.94 (-2.04, -1.83) | 2867766 (2189016,3540798) | 1525817 (992301,1940861) | 462.59 (353.1,571.15) | 231.83 (150.77,294.89) | -1.93 (-2.04, -1.82) |
| 5-9 | 3603 (2790,4358) | 2014 (1451,2495) | 0.62 (0.48,0.75) | 0.29 (0.21,0.36) | -2.08 (-2.22, -1.94) | 302727 (234748,365071) | 170841 (124701,210810) | 51.88 (40.23,62.56) | 24.87 (18.15,30.68) | -2.05 (-2.19, -1.92) |
| 10-14 | 2392 (1903,3184) | 1944 (1434,2538) | 0.45 (0.36,0.59) | 0.29 (0.22,0.38) | -1.13 (-1.23, -1.03) | 189216 (151493,251317) | 154986 (114862,201505) | 35.32 (28.28,46.92) | 23.25 (17.23,30.23) | -1.11 (-1.21, -1.01) |
| 15-19 | 2425 (1896,3292) | 1848 (1520,2492) | 0.47 (0.36,0.63) | 0.3 (0.24,0.4) | -1.6 (-1.67, -1.53) | 179809 (142094,243309) | 138661 (115160,185092) | 34.62 (27.36,46.84) | 22.22 (18.46,29.66) | -1.56 (-1.62, -1.49) |
| 20-24 | 3532 (2788,4978) | 3047 (2484,3831) | 0.72 (0.57,1.01) | 0.51 (0.42,0.64) | -1.38 (-1.54, -1.22) | 243235 (192870,340007) | 211425 (172545,264987) | 49.43 (39.19,69.09) | 35.41 (28.89,44.37) | -1.35 (-1.5, -1.19) |
| 25-29 | 3191 (2585,4190) | 2978 (2490,3636) | 0.72 (0.58,0.95) | 0.51 (0.42,0.62) | -1.28 (-1.43, -1.12) | 204814 (166522,267533) | 192707 (162629,236080) | 46.27 (37.62,60.44) | 32.75 (27.64,40.13) | -1.24 (-1.39, -1.09) |
| 30-34 | 2728 (2200,3622) | 2824 (2415,3464) | 0.71 (0.57,0.94) | 0.47 (0.4,0.57) | -1.38 (-1.45, -1.31) | 162289 (131757,215755) | 170039 (146698,207615) | 42.11 (34.19,55.98) | 28.13 (24.27,34.35) | -1.32 (-1.39, -1.26) |
| 35-39 | 3241 (2612,4160) | 3462 (2851,4259) | 0.92 (0.74,1.18) | 0.62 (0.51,0.76) | -1.26 (-1.32, -1.2) | 176076 (141412,224950) | 190469 (156970,232281) | 49.99 (40.15,63.86) | 33.96 (27.99,41.41) | -1.21 (-1.26, -1.15) |
| 40-44 | 3376 (2752,4250) | 3921 (3245,4574) | 1.18 (0.96,1.48) | 0.78 (0.65,0.91) | -1.47 (-1.63, -1.3) | 166282 (136874,208169) | 195475 (163871,226242) | 58.04 (47.78,72.66) | 39.08 (32.76,45.23) | -1.42 (-1.57, -1.26) |
| 45-49 | 3511 (2910,4597) | 4490 (3805,5223) | 1.51 (1.25,1.98) | 0.95 (0.8,1.1) | -1.62 (-1.77, -1.48) | 154979 (129037,202385) | 200855 (171331,232875) | 66.74 (55.57,87.16) | 42.42 (36.18,49.18) | -1.56 (-1.69, -1.43) |
| 50-54 | 3965 (3285,5015) | 4885 (4196,5519) | 1.87 (1.55,2.36) | 1.1 (0.94,1.24) | -1.83 (-2.01, -1.65) | 155802 (130063,195900) | 195176 (167517,221356) | 73.29 (61.19,92.16) | 43.87 (37.65,49.75) | -1.74 (-1.91, -1.57) |
| 55-59 | 4974 (4156,6412) | 6756 (5761,7730) | 2.69 (2.24,3.46) | 1.71 (1.46,1.95) | -1.54 (-1.75, -1.32) | 171109 (142802,219264) | 236150 (200971,269497) | 92.39 (77.11,118.39) | 59.67 (50.79,68.1) | -1.44 (-1.63, -1.24) |
| 60-64 | 6940 (5953,8939) | 9507 (8018,10799) | 4.32 (3.71,5.57) | 2.97 (2.51,3.37) | -1.43 (-1.55, -1.3) | 205463 (176078,264419) | 285683 (243513,321785) | 127.93 (109.63,164.64) | 89.26 (76.09,100.54) | -1.33 (-1.44, -1.22) |
| 65-69 | 7511 (6396,9936) | 11677 (9881,12962) | 6.08 (5.17,8.04) | 4.23 (3.58,4.7) | -1.14 (-1.24, -1.04) | 187637 (160279,248037) | 295250 (251943,328085) | 151.8 (129.67,200.66) | 107.04 (91.34,118.94) | -1.04 (-1.13, -0.95) |
| 70-74 | 6710 (5773,8782) | 11863 (10282,13249) | 7.93 (6.82,10.37) | 5.76 (5,6.44) | -1 (-1.13, -0.87) | 138543 (119725,180308) | 248620 (216854,276486) | 163.64 (141.42,212.98) | 120.78 (105.35,134.32) | -0.89 (-1.02, -0.76) |
| 75-79 | 6644 (5867,8415) | 10585 (9314,11678) | 10.79 (9.53,13.67) | 8.03 (7.06,8.85) | -1.03 (-1.21, -0.85) | 109867 (96828,138581) | 177370 (156204,196527) | 178.48 (157.3,225.13) | 134.49 (118.44,149.01) | -0.93 (-1.1, -0.77) |
| 80-84 | 5385 (4735,6645) | 9874 (8585,10945) | 15.22 (13.38,18.78) | 11.27 (9.8,12.5) | -0.94 (-1.15, -0.73) | 69698 (61752,85695) | 128347 (112104,141313) | 197.02 (174.56,242.24) | 146.54 (128,161.35) | -0.88 (-1.06, -0.7) |
| 85-89 | 3980 (3453,4675) | 7564 (6379,8344) | 26.34 (22.85,30.93) | 16.54 (13.95,18.25) | -1.44 (-1.75, -1.13) | 40622 (35545,47692) | 77333 (65841,85089) | 268.82 (235.23,315.61) | 169.14 (144,186.1) | -1.4 (-1.67, -1.13) |
| 90-94 | 1663 (1382,1920) | 4230 (3416,4731) | 38.8 (32.24,44.81) | 23.65 (19.09,26.44) | -1.63 (-1.98, -1.28) | 14719 (12260,16981) | 37494 (30663,41839) | 343.49 (286.11,396.27) | 209.59 (171.4,233.88) | -1.61 (-1.93, -1.29) |
| 95+ | 519 (404,598) | 1720 (1291,1955) | 50.99 (39.7,58.76) | 31.56 (23.69,35.88) | -1.71 (-2.1, -1.32) | 4347 (3430,4994) | 14283 (10825,16222) | 426.94 (336.92,490.51) | 262.06 (198.62,297.64) | -1.75 (-2.13, -1.37) |

**EAPC Estimated Annual Percentage Change, DALYs Disability-Adjusted Life Years, ASR Age-Standardized Rate, UI uncertainty interval, CI confidence interval.**

**Table S3 Projection of the Global Burden of Adverse effects of medical treatment for the Next 15 Years Based on the BAPC Model.**

| **Year** | **Incidence** | | **Prevalence** | | **Death** | | **DALYs** | |
| --- | --- | --- | --- | --- | --- | --- | --- | --- |
|  | **Absolute numbers (95%UI)** | **ASR (per 100,000, 95%UI)** | **Absolute numbers (95%UI)** | **ASR (per 100,000, 95%UI)** | **Absolute numbers (95%UI)** | **ASR (per 100,000, 95%UI)** | **Absolute numbers (95%UI)** | **ASR in (per 100,000, 95%UI)** |
| **2022** | **11444193 (10667035,12221352)** | **142.25 (132.59,151.9)** | **873370 (814524,932216)** | **10.86 (10.12,11.59)** | **120212 (116434,123991)** | **1.49 (1.45,1.54)** | **4971409 (4769600,5173217)** | **61.79 (59.28,64.3)** |
| **2023** | **11030336 (10038428,12022244)** | **135.79 (123.58,148.01)** | **841847 (766512,917183)** | **10.36 (9.44,11.29)** | **118546 (113067,124025)** | **1.46 (1.39,1.53)** | **4876302 (4612317,5140286)** | **60.03 (56.78,63.28)** |
| **2024** | **10625250 (9363823,11886677)** | **129.6 (114.21,144.98)** | **810989 (714979,906999)** | **9.89 (8.72,11.06)** | **116852 (109218,124486)** | **1.43 (1.33,1.52)** | **4781324 (4434939,5127709)** | **58.32 (54.09,62.54)** |
| **2025** | **10228949 (8666584,11791315)** | **123.64 (104.76,142.53)** | **780797 (661717,899878)** | **9.44 (8,10.88)** | **115135 (105043,125227)** | **1.39 (1.27,1.51)** | **4686415 (4244104,5128727)** | **56.65 (51.3,61.99)** |
| **2026** | **9842080 (7962060,11722100)** | **117.93 (95.4,140.46)** | **751323 (607897,894748)** | **9 (7.28,10.72)** | **113400 (100630,126171)** | **1.36 (1.21,1.51)** | **4591769 (4044228,5139310)** | **55.02 (48.46,61.58)** |
| **2027** | **9464381 (7259649,11669113)** | **112.45 (86.25,138.64)** | **722545 (554235,890854)** | **8.58 (6.59,10.58)** | **111653 (96032,127274)** | **1.33 (1.14,1.51)** | **4497500 (3838112,5156889)** | **53.44 (45.6,61.27)** |
| **2028** | **9095992 (6566315,11625670)** | **107.19 (77.38,137)** | **694474 (501261,887686)** | **8.18 (5.91,10.46)** | **109892 (91291,128493)** | **1.3 (1.08,1.51)** | **4403637 (3627841,5179434)** | **51.89 (42.75,61.04)** |
| **2029** | **8736859 (5886876,11586842)** | **102.15 (68.83,135.47)** | **667105 (449342,884869)** | **7.8 (5.25,10.35)** | **108115 (86436,129793)** | **1.26 (1.01,1.52)** | **4310247 (3414969,5205525)** | **50.39 (39.93,60.86)** |
| **2030** | **8387036 (5224936,11549136)** | **97.31 (60.62,134)** | **640444 (398754,882134)** | **7.43 (4.63,10.23)** | **106328 (81497,131158)** | **1.23 (0.95,1.52)** | **4217361 (3200651,5234072)** | **48.93 (37.13,60.73)** |
| **2031** | **8047008 (4583464,11510552)** | **92.67 (52.79,132.56)** | **614527 (349723,879331)** | **7.08 (4.03,10.13)** | **104539 (76499,132579)** | **1.2 (0.88,1.53)** | **4125233 (2985949,5264517)** | **47.51 (34.39,60.63)** |
| **2032** | **7716564 (3964387,11468742)** | **88.24 (45.33,131.14)** | **589339 (302398,876280)** | **6.74 (3.46,10.02)** | **102753 (71460,134046)** | **1.17 (0.82,1.53)** | **4033957 (2771639,5296275)** | **46.13 (31.69,60.56)** |
| **2033** | **7395945 (3369569,11422321)** | **83.99 (38.27,129.72)** | **564898 (256921,872875)** | **6.42 (2.92,9.91)** | **100970 (66398,135542)** | **1.15 (0.75,1.54)** | **3943550 (2558486,5328613)** | **44.78 (29.06,60.51)** |
| **2034** | **7085163 (2800252,11370073)** | **79.93 (31.59,128.27)** | **541205 (213388,869021)** | **6.11 (2.41,9.8)** | **99191 (61330,137052)** | **1.12 (0.69,1.55)** | **3854096 (2347168,5361025)** | **43.48 (26.48,60.48)** |
| **2035** | **6784156 (2257248,11311064)** | **76.04 (25.3,126.79)** | **518254 (171861,864647)** | **5.81 (1.93,9.69)** | **97419 (56270,138568)** | **1.09 (0.63,1.55)** | **3765595 (2138182,5393009)** | **42.21 (23.97,60.45)** |
| **2036** | **6493037 (1741113,11244960)** | **72.33 (19.4,125.27)** | **496056 (132384,859728)** | **5.53 (1.47,9.58)** | **95663 (51233,140094)** | **1.07 (0.57,1.56)** | **3678228 (1932028,5424428)** | **40.98 (21.52,60.43)** |

**DALYs Disability-Adjusted Life Years, ASR Age-Standardized Rate, UI uncertainty interval.**

**Table S4 The Slope Index of Inequality and the Concentration Index for Adverse effects of medical treatment from 1990 to 2021.**

|  |  | **Incidence** | **Prevalence** | **Death** | **DALYs** |
| --- | --- | --- | --- | --- | --- |
| **Slope Index of Inequality (95% CI)** | **1990** | **91.410(64.967, 117.853)** | **6.983(4.962, 9.003)** | **-3.432(-4.189, -2.675)** | **-200.693(-224.354, -177.031)** |
|  | **2021** | **98.728(77.706, 119.750)** | **7.542(5.936, 9.149)** | **-2.396(-2.876, -1.916)** | **-101.395(-116.741, -86.049)** |
| **Concentration Index (95% CI)** | **1990** | **-0.329(-0.396, -0.264)** | **-0.329(-0.395, -0.264)** | **0.255(0.169, 0.332)** | **0.373(0.286, 0.452)** |
|  | **2021** | **-0.368(-0.440, -0.300)** | **-0.367(-0.440, -0.299)** | **0.199(0.112, 0.278)** | **0.349(0.260, 0.425)** |

**DALYs Disability-Adjusted Life Years, CI confidence interval.**
